# Neurofilament light and tau as blood biomarkers for sports-related concussion

**DOI:** 10.1212/WNL.0000000000005518

**Published:** 2018-05-15

**Authors:** Pashtun Shahim, Yelverton Tegner, Niklas Marklund, Kaj Blennow, Henrik Zetterberg

**Affiliations:** From the Institute of Neuroscience and Physiology, Department of Psychiatry and Neurochemistry (P.S., K.B., H.Z.), the Sahlgrenska Academy at University of Gothenburg; Clinical Neurochemistry Laboratory (P.S., K.B., H.Z.), Sahlgrenska University Hospital, Mölndal; Division of Medical Sciences, Department of Health Sciences (P.S.), Luleå University of Technology; Department of Clinical Sciences (Y.T.), Skåne University Hospital, Lund University, Sweden; Department of Neurology (N.M.), Washington University School of Medicine, St. Louis, MO; Department of Molecular Neuroscience (H.Z.), UCL Institute of Neurology, Queen Square; and UK Dementia Research Institute (H.Z.), London.

## Abstract

**Objective:**

To compare neurofilament light (NfL) and tau as blood-based biomarkers for acute sports-related concussion (SRC) and determine whether their concentrations at different time points after the injury are associated with prolonged time to return to play (RTP).

**Methods:**

A total of 288 professional hockey players were followed longitudinally from September 1, 2012, to April 30, 2015. Data collection and biomarker analyses were conducted between 2015 and 2017. Associations were tested between blood concentrations of NfL and tau, and RTP time. Serum concentrations of S100B and neuron-specific enolase (NSE) were also measured for comparison.

**Results:**

Of 288 players, 105 sustained an SRC. Of these, 87 underwent blood sampling 1, 12, 36, and 144 hours after SRC and at the RTP time point. Serum NfL concentrations 1, 12, 36, and 144 hours after SRC were related to prolonged RTP time, and could separate players with RTP >10 days from those with RTP ≤10 days (area under the receiver operating characteristic curve [AUROC] 0.82). Also, serum NfL 144 hours after SRC discriminated players who resigned from the game due to persistent postconcussion symptoms (PCS) from those who returned to play (AUROC 0.89). Plasma tau 1 hour after SRC was related to RTP but less strongly than NfL, while S100B and NSE showed no such associations.

**Conclusion:**

Serum NfL outperformed tau, S100B, and NSE as a biomarker for SRC. From a clinical standpoint, serum NfL may be useful to identify individuals at risk of prolonged PCS, and may aid in biomarker-informed decisions with regard to when RTP should be considered.

Sports-related concussion (SRC) is a major health concern in the United States, with an estimated 1.6–3.6 million cases occurring annually.^1^ Although most concussed athletes recover and return to play (RTP) within days to weeks, a subset of these have neurobehavioral symptoms that may persist for months to years.^2^ Determining when it may be considered safe to RTP is essential, as athletes with premature RTP may be at increased risk of developing persistent postconcussion symptoms (PCS), especially if another concussion is sustained.^3–5^ Thus, objective biomarkers that can monitor the course of recovery in concussed athletes would be a major contribution to the field.

Axonal injury, particularly injury to white matter axons, has been hypothesized to be the key type of damage and the primary determinant of outcome following traumatic brain injury (TBI).^6–9^ Neurofilament light (NfL) and tau are axonal proteins that have been reported to increase in CSF of individuals with both mild and severe TBI.^5,8,10^ We recently developed an immunoassay on the ultrasensitive Single molecule assay (Simoa) platform for quantification of NfL in blood with approximately 100-fold greater analytical sensitivity compared with a standard immunoassay, allowing measurement of NfL in all samples.^11–13^ In the context of SRC, we observed increased concentrations of serum NfL in boxers who received repetitive punches to the head.^11^ In regards to plasma tau, also quantified by Simoa, we observed increased levels in concussed hockey players compared with preseason samples.^14^ Considering these promising isolated findings, we herein compared the performances and temporal dynamics of serum NfL and plasma tau following acute SRC and examined whether increased blood concentrations of these relate to prolonged RTP time in a large cohort of professional hockey players. We also analyzed biomarkers related to astrogliosis (S100 calcium binding protein B [S100B]) and neuronal injury (neuron-specific enolase [NSE]) as these are also implicated in TBI.^15–17^ We hypothesized that serum NfL would be more strongly associated with SRC than tau, S100B, and NSE.

## Methods

### Study population

This is a prospective cohort study of concussion among professional hockey players (n = 288) from 14 teams in the top Swedish Hockey League, who were followed longitudinally from September 1, 2012, to April 1, 2015. Eighty-seven players, who sustained SRC during this period, underwent consecutive blood sampling at 1, 12, 36, and 144 hours after SRC, or the day when the player returned to unrestricted competition (RTP). Players from 4 of the contesting teams were also sampled for baseline values prior to the start of the hockey season (preseason samples). Players from one of the teams were also sampled before (n = 28) and 1 (n = 20) and 12 (n = 19) hours after a friendly game without concussion incidents. We also enrolled 19 neurologically healthy nonathletic controls (HC) without known history of brain trauma or any other neurologic disease as well as 12 noncontact sport athletes, a group of gymnasts (GC), for comparison. Data collection and blood biomarker analyses were conducted between 2015 and 2017. Twenty-eight of the concussed players as well as the preseason values from 2 of the above teams have been previously reported.^14^

### Study protocol

During the preseason, the physicians of all the 14 contesting teams were provided with a concussion kit, containing injury protocol, Rivermead Post-Concussion Symptoms Questionnaire (RPQ),^18^ instructions for blood tests, and blood sampling equipment and tubes. As the teams move across the country for playing matches, we also provided all the local laboratories in the residing cities of each team with instructions to handle blood samples. The teams' physicians were present at all regular season games, documenting signs and symptoms of concussion and physical examination findings in the event of a concussion. The teams' physicians also recorded the date when a player had completely recovered from concussion and was able to return to unrestricted competition.

### Severity of SRC

The severity of SRC was graded according to the latest SRC guidelines, which is based on the number of days it takes for a player to RTP.^19^ The players who displayed clinical signs of concussion during the game were removed from the game and followed a graded RTP protocol.^19^ As approximately half of the athletes returned to play within 10 days after an SRC, we pre hoc defined the following severity categories: (1) RTP ≤10 days and (2) RTP >10 days. Our primary hypothesis was that players with RTP >10 days would have higher concentrations of the biomarkers than players who could RTP within 10 days.

### Biochemical procedures

Blood samples were collected by venipuncture into gel-separator tubes for serum and ethylenediaminetetraacetic acid tubes for plasma and centrifuged within 20–60 minutes. Serum and plasma were separated, aliquoted, and stored at −80°C pending biochemical analysis. Plasma tau was measured with a commercially available immunoassay using digital array technology (Quanterix Corporation, Lexington, MA).

Serum NfL and plasma tau were measured with novel immunoassays using digital array technology (Quanterix Corporation, Lexington, MA).^11,20^ The limit of detection for the NfL assay is 0.29 pg/mL, which is approximately 100-fold more sensitive than conventional assay for the protein, while the limit of detection for tau is 0.02 pg/mL, which is over 1,000-fold more sensitive than conventional immunoassays for the protein. Samples for S100B and NSE were analyzed on a Modular E170 instrument (Roche Diagnostics, Mannheim, Germany) with reagents from the same manufacturer.

All samples were analyzed at the same time using the same batch of reagents by board-certified laboratory technicians who were blind to clinical information. As specified above, 28 of the players and preseason values from 2 of the contesting teams were part of an earlier report from our group,^14^ but their samples were re-analyzed in the current investigation to minimize any influence of batch-related variation on the data.

### Statistical analysis

For comparisons of blood biomarker concentrations vs the preseason levels, the Mann-Whitney *U* test, and for multiple time group comparison, the Kruskal-Wallis one-way analysis of variance were used. The Spearman rank correlation coefficient (ρ) was used for analyses of correlation between biomarkers and age. To examine the potential influence of age on the results, we also performed analysis of covariance of log-transformed continuous data with and without age as a covariate. Partial correlation examined the associations between blood biomarkers and RPQ scores adjusted for age. Logistic regression models were constructed with the RTP duration dichotomized (RTP >10 days vs RTP ≤10 days) as dependent variable and biomarker as independent, covaried for age. Continuous variables (age and biomarker concentrations) in the logistic regression models were standardized (x−μ/σ; where x is a raw/biomarker score, μ is the mean, and σ is the SD) to facilitate comparisons between different biomarkers. The area under the receiver operating characteristic curve (AUROC) was calculated for determining the prognostic accuracy of the biomarkers.

All tests were 2-sided and statistical significance was determined at *p* < 0.05. Correction for multiple group comparison was done using the Dunn or Turkey test. All statistical calculations were performed using GraphPad Prism 6.0 (GraphPad Inc., San Diego, CA) and R (v. 3.2.3, The R Foundation for Statistical Computing).

### Standard protocol approvals, registrations, and patient consents

The Ethics Committee for Medical Research at the University of Gothenburg, Sweden, and the Swedish Hockey Association approved the study. Written informed consent was obtained from all participants.

### Data availability

The raw data used in preparation of the figures and tables will be made available or shared in anonymized format by request of a qualified investigator.

## Results

**Figure 1:**
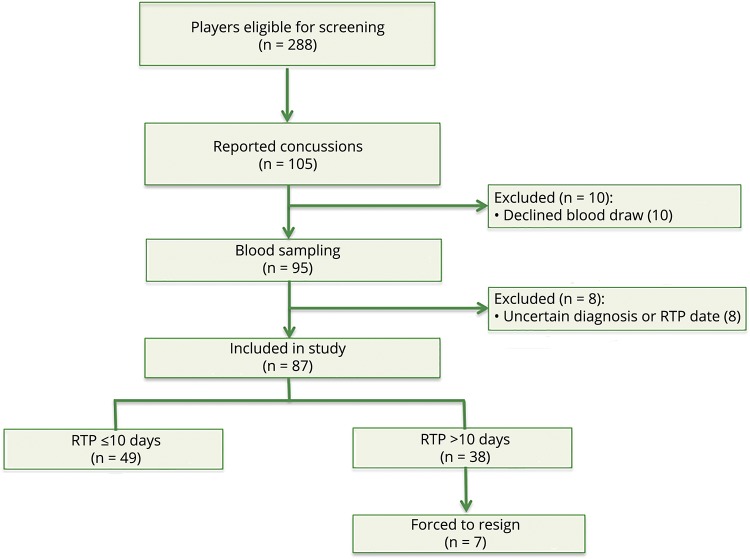
Screening and enrollment of study participants RTP = return to play.

**Figure 2:**
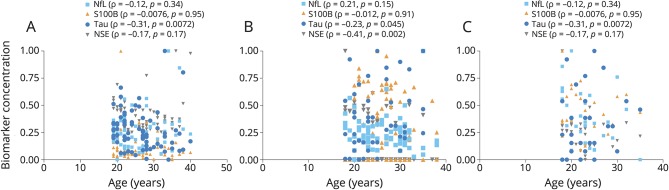
Effect of age on the blood-based biomarkers (A–C) Correlation between age and blood-based biomarkers in concussed athletes and preseason sample controls (both healthy controls and gymnasts). The *ρ* and *p* value are from the Spearman rank correlation. NfL = neurofilament light; NSE = neuron-specific enolase; S100B = S100 calcium-binding protein.

A total of 288 professional hockey players were followed from August 1, 2012, to April 1, 2015. Of 288 players who were followed for 3 years, 105 players were reported to have sustained a SRC. Of these, 95 players consented to undergo repeated blood sampling. For 8 of these players, there were uncertainties regarding the diagnosis or RTP date, which left 87 players in the final study cohort (figure 1). Forty-nine of the 87 players had RTP ≤10 days, while 38 had RTP >10 days (figure 1). Among players with RTP >10 days, 7 had persistent PCS for more than a year and eventually resigned from the game. The demographic and clinical characteristics of the study participants are summarized in table 1. There was no difference in age between the concussed players and the group of players who contributed preseason samples or the HC (*p* = 0.30 and *p* = 0.99, respectively, table 1). However, there was a difference in age between the SRC group and GC (*p* < 0.001). There was no association between age and any biomarker concentration in concussed players except for tau (Spearman *ρ* = −0.31, *p* = 0.0072; figure 2A). Plasma tau also correlated with age in players who contributed preseason samples and in HC (figure 2, B–C). There was also a correlation between age and NSE in players who contributed preseason samples (figure 2B).

**Table 1:**
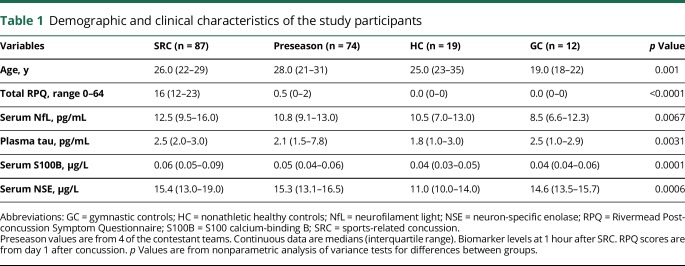
Demographic and clinical characteristics of the study participants

### Blood-based biomarkers across different control groups

There was no significant difference in the concentration of serum NfL and plasma tau between the HC, GC, or preseason (*p* = 0.20 and *p* = 0.091, respectively; table 1). In contrast, serum concentrations of S100B and NSE were increased in preseason samples compared with HC and GC (*p* < 0.001 and *p* < 0.0001, respectively; table 1).

### Blood NfL and tau changes following SRC

**Figure 3:**
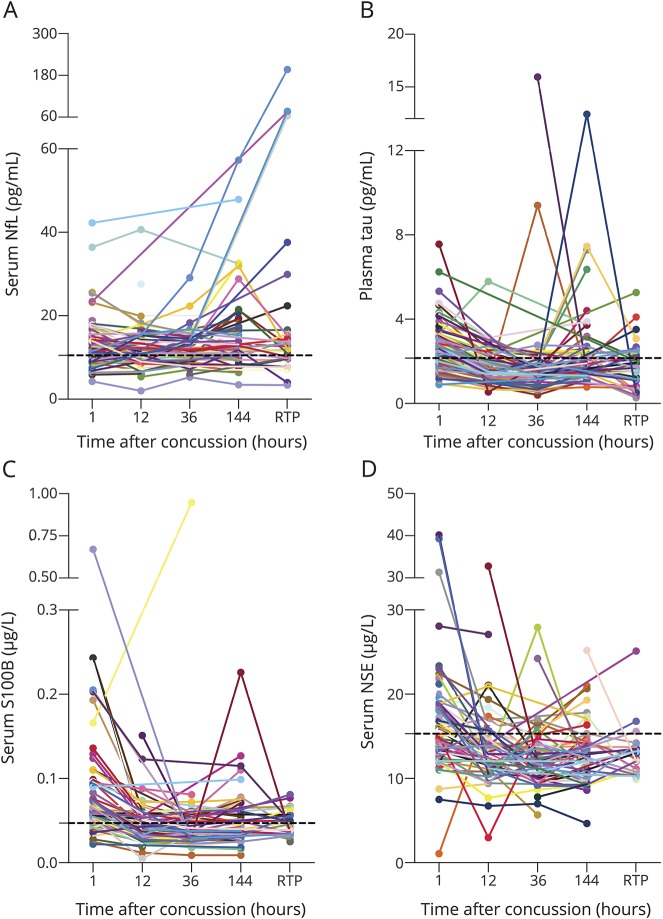
Temporal profile of the blood biomarkers in concussed professional hockey players (A–D) Dynamic of blood neurofilament light (NfL), tau, S100 calcium-binding protein B (S100B), and neuron-specific enolase (NSE) in concussed professional hockey players measured at 1, 12, 36, and 144 hours, and when the players returned to play. The black dotted lines in each plot show the median of the preseason samples.

Serum NfL concentrations in samples collected 1 hour after SRC were higher than in preseason, HC, and GC samples (*p* = 0.020, *p* = 0.030, and *p* = 0.010, respectively; table 1). Figure 3A shows the time course of serum NfL in concussed players (*F*_1,242_ = 69, *p* < 0.0001). After an initial increase at the 1-hour time point, serum NfL concentration dropped at 12 hours and increased again in a steady fashion, with the highest concentrations in samples collected 10 days after injury (*p* < 0.0001, compared to all time points).

Plasma tau concentration 1 hour after SRC was higher than in the group of hockey players who contributed samples prior to the start of the season (*p* = 0.050; table 1). A similar result was obtained when compared with the HC, but not compared with GC (*p* = 0.0003, and *p =* 0.80, respectively; table 1). Figure 3B shows the time course of plasma tau in concussed players (*F*_1,257_ = 1929, *p* < 0.0001), where the concentrations increased 1 hour after SRC and dropped at the 12-hour time point (*p* < 0.0001), but rose again at 144 hours (*p* < 0.0001), whereafter the levels normalized at RTP.

### Serum S100B and NSE changes following SRC

Serum S100B concentration was elevated 1 hour after SRC compared with preseason, HC, and GC (*p* = 0.0020, *p <* 0.0001, and *p* = 0.014, respectively; table 1). Also, serum NSE concentrations increased 1 hour after SRC compared with the HC, but not compared with GC or preseason samples (*p* = 0.0020, *p* = 0.80, and *p* = 0.55, respectively; table 1). Figure 3, C–D, shows the time course of serum S100B. The highest levels of both S100B and NSE were seen 1 hour after SRC, whereafter they rapidly decreased (*F*_1,246_ = 397, *p* < 0.0001 and *F*_1,250_ = 314, *p* < 0.0001 for S100B and NSE, respectively).

### Association between blood biomarkers and symptom severity

Concussed players had increased RPQ scores 1 hour after SRC (median 16, interquartile range [IQR] 12–23) compared with baseline (median 0.5, IQR 0.0–2.0) and HC (0.0, 16, IQR 0.0–0.0; *p* < 0.0001). Both NfL and tau at the 1-hour sampling time point correlated with RPQ scores (*ρ* = 0.41, *p* = 0.011, and *ρ* = 0.32, *p* = 0.056, respectively). There was no correlation between the concentrations of S100B or NSE and RPQ scores (*ρ =*0.12, *p* = 0.51, and *ρ* = −0.017, *p* = 0.92, respectively).

### Biomarker concentrations in relation to RTP

**Figure 4:**
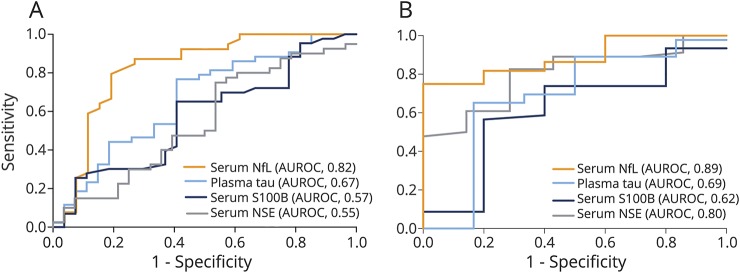
Prognostic utility of the biomarkers at selected time points after sports-related concussion (SRC) (A) Area under the receiver operating characteristic curve (AUROC) for the biomarkers at 1 hour after SRC in players with return to play (RTP) >10 days vs RTP ≤10 days. (B) AUROC for the biomarkers at 144 hours after SRC in players with persistent postconcussion symptoms who eventually resigned vs players who could RTP. NfL = neurofilament light; NSE = neuron-specific enolase; S100B = S100 calcium-binding protein B.

Serum NfL concentrations were increased in players with RTP >10 days compared with players with RTP ≤10 days at all measured time points (table 2). Serum NfL 1 hour after SRC could separate players with RTP >10 days from RTP ≤10 days with an AUROC of 0.82 (*p* < 0.0001; figure 4A). The AUROC for serum NfL remained high also at 12, 36, and 144 hour time points (0.72, 0.73, and 0.73, respectively; table 2). In players who resigned from the game, serum NfL rose continuously over the course of 144 hours compared with players who could return to play, where the levels normalized in almost all at RTP. Serum NfL concentrations at the 144-hour time point could separate players who resigned from the game from those who could RTP with an AUROC of 0.89 (*p* = 0.0050; figure 4A). Also, plasma tau concentrations 1 hour after SRC were increased in players with RTP >10 days vs RTP ≤10 days; however, with limited prognostic utility (AUROC 0.67; table 2). Plasma tau concentrations beyond the 1-hour time point were not associated with duration of RTP (table 2). In contrast, the other biomarkers performed poorly in separating players with RTP >10 days from RTP ≤10 days (figure 4A and table 2). Also, the performance of tau, S100B, and NSE was inferior to NfL in separating players with persistent PCS who resigned from the game from those who could RTP (figure 4B).

**Table 2:**
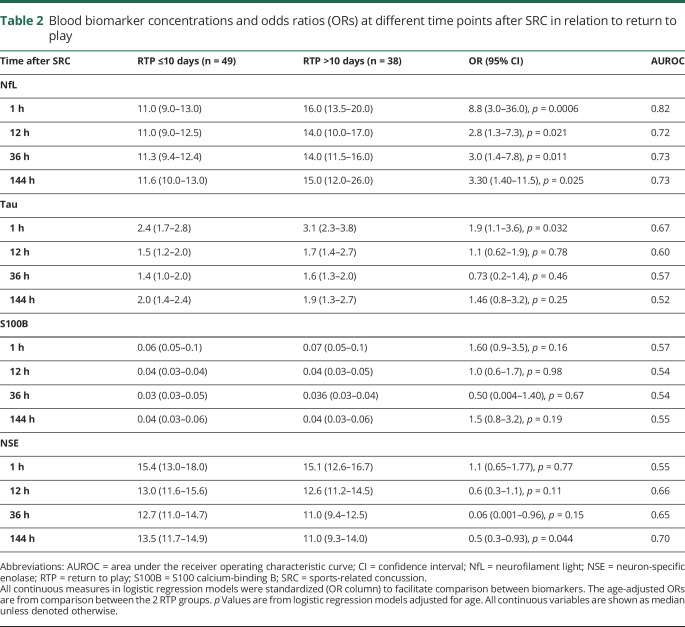
Blood biomarker concentrations and odds ratios (ORs) at different time points after SRC in relation to return to play

### Biomarker concentrations after a game without concussion

Finally, to assess the effect of physical exertion on the blood biomarker levels, players from one of the teams were sampled prior to and 1 hour after a friendly game of hockey. The concentrations of all the studied biomarkers, except for NfL, increased 1 hour after a game of hockey without concussion compared with levels prior to the game, and the levels normalized at 12 hours after the game (table 3).

**Table 3:**
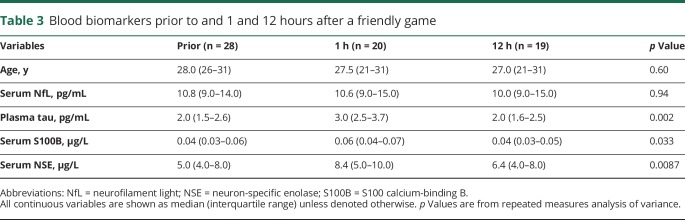
Blood biomarkers prior to and 1 and 12 hours after a friendly game

## Discussion

In this study, we compared NfL head-to-head with tau, S100B, and NSE as blood-based biomarkers for SRC and assessed their relationship to duration of RTP in a large prospective cohort of professional hockey players. The main findings were that (1) serum NfL concentrations were increased 1, 12, 36, and 144 hours after SRC compared with preseason concentrations with higher concentrations in athletes with RTP >10 days, (2) initial NfL concentrations correlated with RPQ scores, and (3) serum NfL was the only biomarker that remained elevated over time in the subset of players with persistent PCS over 1 year who eventually resigned from the game. In addition, we found that plasma tau concentration 1 hour after SRC increased compared with preseason levels; elevated levels at the 1-hour time point were related to RTP but with limited prognostic utility. Both S100B and NSE increased 1 hour after SRC but without any correlations with injury severity or duration of RTP. Finally, physical exercise may be a confounder for all biomarkers except NfL.

A major challenge in the field of concussion has been developing conventional biomarkers that could identify individuals at risk of developing long-term or chronic PCS. Experimental models link insufficient recovery or unresolved concussion with greater neuropathology.^3,4^ Axonal injury, including redistribution of neurofilament proteins, has been hypothesized to be a central mechanism of TBI, and a major determinant of outcome following TBI.^7,8,21^ Previous studies measuring serum NfL in samples from patients with severe TBI as well as neurodegenerative diseases have shown tight correlations with corresponding CSF samples, suggesting that serum NfL reflects axonal damage in the brain.^11,12,22^ In the present study, serum NfL measured by Simoa increased in professional hockey players immediately after SRC, with the highest levels measured 144 hours after injury, and the levels normalized in players who returned to play. Also, initial levels of serum NfL correlated with RPQ scores, and elevated serum NfL concentrations 1–144 hours after SRC were related to duration of RTP. These results corroborate the findings of a recent study reporting increased CSF and serum NfL in amateur boxers who received repetitive punches to the head, but were not knocked out.^23^ In addition, in the subset of players who resigned from the game due to persistent PCS, we observed a sustained release of NfL over 144 hours. These findings are in agreement with a recent study of persistent PCS in professional hockey players, where the levels of NfL were elevated in CSF months to years after recent SRC.^5^ Taken together, these results suggest that serum NfL analyzed 1–144 hours after SRC may be useful to identify individuals at risk of prolonged PCS, and may aid in guiding objective biomarker-based informed decisions with regard to RTP.

In recent years, a number of isolated and rather small studies have found increased plasma tau after SRC.^14,24,25^ Consistently, we also observed increased plasma tau 1 hour after SRC compared with preseason samples. However, in comparison to serum NfL, tau showed inferior diagnostic and prognostic utility at all the measured time points in the present study. A previous smaller study of plasma tau in college athletes reported increased concentrations of tau 1 and 72 hours after SRC in the subset of athletes with RTP >10 days compared with those with RTP ≤10 days,^24^ a result that we partially failed to replicate in this larger study. A possible explanation could be that determining RTP in professional athletes is more challenging due to other factors not present in amateurs that may influence such decisions. Also, plasma tau seems to be sensitive to physical exertion, and professional athletes often follow a strict protocol of physical training after 24 hours of rest.^19^ In addition, we found no difference in the concentrations of plasma tau between players with persistent PCS who resigned from the game compared with those who could RTP, while serum NfL was highly prognostic. These findings are in line with NfL and tau measured in the CSF of these players, where their CSF tau was unaltered, while CSF NfL was elevated.^5^ Similar findings were also observed when NfL and tau were measured in CSF of amateur boxers who received repetitive punches to the head, with CSF tau showing inferior correlation to the number of punches or severity of head injury compared to CSF NfL.^8^

Similar to plasma tau, the concentrations of S100B and NSE increased 1 hour after SRC and the levels normalized at the 12-hour time point. However, neither S100B nor NSE was associated with the duration of RTP or RPQ scores. These findings are in agreement with previous studies showing increased concentrations after mild TBI, but with limited relation to outcome.^14,26^ A crucial aspect of blood-based biomarkers for SRC is insensitivity to peripheral/body trauma or physical exertion. In the present study, all the measured biomarkers, except NfL, increased in players after a friendly game without head injury. In the case of plasma tau, it seems that athletes as well as military personnel with a history of blast-related concussion may have sustained increased plasma tau in the absence of physical exertion.^24,27^ As for S100B and NSE, several studies have found increased serum S100B and NSE levels in the absence of head injury in soccer players, marathon runners, and patients with peripheral trauma.^28–30^ Consistently, serum NSE concentrations were lower in players who resigned compared with players who could RTP (AUROC 0.80), which could be due to less influence of physical exertion in the former subgroup.^19^ Serum NSE concentration was also increased in GC without head trauma compared with HC, adding further evidence that serum NSE is sensitive to physical exertion. It is also worth mentioning that both tau and NSE concentrations inversely correlated with age, which thus should be taken into account when interpreting results from these markers. Together, these findings argue against tau, NSE, and S100B as blood-based biomarkers for SRC, while the performance of serum NfL was robust over the course of 6 days post SRC without being sensitive to physical exertion.

The main limitation of this study is the lack of individual baseline samples on all of the players, which precludes monitoring biomarker changes preinjury and postinjury within players. Also, our follow-up was not long; longitudinal studies with follow-up times over many years are required to address the risk or protective factors to sustain concussion or recovery.

NfL outperformed tau as well as S100B and NSE as a blood-based biomarker for acute SRC. Integrating measurement of serum NfL in the clinical evaluation of concussed athletes may aid in objectively identifying and assessing those at increased risk of poor recovery to help prevent the development of long-term PCS.

## Glossary

AUROC: area under the receiver operating characteristic curve
GC: gymnast controls
HC: healthy nonathletic controls
IQR: interquartile range
NfL: neurofilament light
NSE: neuron-specific enolase
PCS: postconcussion symptoms
RPQ: Rivermead Post-Concussion Symptoms Questionnaire
RTP: return to play
SRC: sports-related concussion
TBI: traumatic brain injury
